# Cold-Pressing Olive Oil in the Presence of Cryomacerated Leaves of *Olea* or *Citrus*: Nutraceutical and Sensorial Features

**DOI:** 10.3390/molecules24142625

**Published:** 2019-07-19

**Authors:** Chiara Sanmartin, Isabella Taglieri, Monica Macaluso, Cristina Sgherri, Roberta Ascrizzi, Guido Flamini, Francesca Venturi, Mike Frank Quartacci, François Luro, Franck Curk, Luisa Pistelli, Angela Zinnai

**Affiliations:** 1 Department of Agriculture, Food and Environment, University of Pisa, Via del Borghetto 80, 56124 Pisa, Italy; 2 Interdepartmental Research Center “Nutraceuticals and Food for Health”, University of Pisa, Via del Borghetto, 80, 56124 Pisa, Italy; 3 Department of Pharmacy, University of Pisa, Via Bonanno Pisano 6, 56126 Pisa, Italy; 4 UMR AGAP Corse, Station INRA/CIRAD, 20230 San-Giuliano, France

**Keywords:** *Olea europaea* L., *Citrus limon* (L.) Osbeck., *Citrus × aurantium* L., olive oil, phenolic compounds, antioxidants, bioactive compounds, sensory profiles, SPME/GC-MS

## Abstract

The nutraceutical properties of extra-virgin olive oil (EVOO) can be further improved by the addition of olive leaves during olive pressing. However, while *Citrus* leaves are rich sources of bioactive substances, no data are available in the literature about the effect of *Citrus* leaf addition on the nutraceutical and sensorial profiles of olive oil. This study aimed at comparing the chemical and sensorial qualities of olive oils obtained from ripe olives pressed together with either *Olea* or *Citrus* spp. (lemon or orange) cryomacerated leaves. General composition parameters as well as major antioxidants and antioxidant activity were measured. A panel test evaluation, as well as headspace volatile characterization (headspace solid phase microextraction, HS-SPME), were also performed. All data were compared with an EVOO extracted from the same olive batch used as control. It was possible to obtain Leaf Olive Oils (LOOs) characterized by a higher (*p* < 0.05) content of antioxidants, compared to the control sample, and the highest oleuropein concentration was detected in the olive oil extracted in presence of olive leaf (+50% in comparison with the control). All the LOOs showed a higher smell complexity and the scent of ripe fruit was generally mitigated. Lemon and olive LOOs showed the best smell profile.

## 1. Introduction

Across cultures, there are many different dietary patterns, some of which promote health whereas others increase risk of chronic diseases [1]. In particular, people from the Mediterranean area were found to be less susceptible to develop cardiovascular diseases such as hypertension, stroke, and hyperlipidemia than people from North America and North Europe [2,3]. The Mediterranean-style diet is not a specific diet, but rather a collection of dietary habits characterized by a high consumption of fruit, vegetables, legumes, and complex carbohydrates, with a moderate amount of fish, the use of olive oil as the main source of fats, and a low-to-moderate amount of red wine during meals [4]. The notable positive healthy effect of the Mediterranean diet has also been attributed to olive oil, which is one of the most appreciated fats worldwide [5].

The popularity of extra-virgin olive oil relies on its pleasant flavor, due to its volatile compounds and healthy properties, attributed to the phenolic compounds also responsible for bitterness and pungency [6,7]. This pleasantness to the mouth is coupled with a healthy fatty acid composition, characterized by a high content of oleic acid and a richness in bioactive compounds, including polyphenols, responsible for its protective roles, such as antiatherogenic, anti-inflammatory, antiaging, antitumor, antiviral, and immunomodulating activities [5,8,9,10,11,12,13]. Nevertheless, the quality profile of olive oil is easily perishable [14,15] because of its high concentration of unsaturated fatty acids; consequently, natural antioxidants could be used as food additives in order to improve its stability [16]. Due to their healthful and nutritional effects, considerable attention has recently been given to the individuation of natural sources of antioxidants, such as olive oil by-products [17,18] and fruit and vegetable skins [19,20,21].

The benefits of olive (*Olea europaea* L.) leaves have been known for centuries, and they were traditionally used to prevent and treat different diseases [22]. Recently, experimental studies have demonstrated that the olive leaf is endowed with antioxidant, hypoglycemic, antihypertensive, antiarrhythmic and antiatherosclerotic effects, besides antimicrobial, antiviral, antitumor, and anti-inflammatory activities [22,23]. Olive leaves and their extracts are associated not only with health and with the treatment of several diseases, but also with food preservation, mainly because of their polyphenols [22,23]. Oleuropein is one of the main phenolic constituents of the olive leaf, followed by hydroxytyrosol, verbascoside, and 7-*O*-glucosides of luteolin and apigenin; these phenols, together with triterpenes, other flavones, and chalcones, are thought to be responsible for the olive leaf’s pharmacological and antioxidant effects [22,23,24,25].

*Citrus* (*Citrus* L., Rutaceae) is one of the most popular world fruit crops and has a strong commercial value, being produced mainly for fresh consumption but also for the production of fruit juice. Moreover, its by-products are a source of important bioactive compounds with potential for animal feed, manufactured foods and health care. [26]. Indeed, citrus fruits such as orange and lemon provide a great number of secondary metabolites, phytochemicals which play a major defensive and attractive role in the interactions between plants and their environment, but also having many interesting applications, for example as medicinal and pharmaceutical raw materials, but also in the manufacture of perfumes, cosmetics and food [27].

It has been reported that citrus extracts exhibit a wide range of promising biological properties, including antiatherogenic, anti-inflammatory, antitumor, anticlotting and antioxidant activities, due to their phenolic profile [28]. Their radical scavenging activity is widely reported in literature and, as well known, reactive oxygen species (ROS) have found to be supportive in the pathogenesis of human beings [28]. *Citrus* leaves in particular are rich sources of bioactive substances, including antioxidants, such as ascorbic acid, flavonoid and phenolic derivatives, which are expected to exert strong antioxidant effects. In this context, they have assumed great importance, especially considering the use of antioxidant plant extracts both as an alternative to food preservation technology and as prophylactic agents for some human diseases [29].

The present work reports the composition of the essential oils extracted from the leaves of orange, lemon and olive; then, it deals with the formulation of three leaf olive oils (LOOs) by means of the addition of citrus and olive leaves to the olives during the oil extraction. Moreover, the panel test evaluation of the three flavored oils, as well as the chemical characterization of their spontaneous volatile emission by means of headspace solid phase micro-extraction (HS-SPME), was performed.

## 2. Results

### 2.1. Essential Oil Compositions

The complete identification of all the essential oils’ (EOs’) compositions is reported in Table 1. Overall, 89 compounds were identified among the extracted EOs, which exhibited compositions with oxygenated monoterpenes as the most abundant class of volatiles, ranging from 25.7% in *Olea europaea* L. leaves up to 81.0% in *Citrus × aurantium* L.

Although the monoterpenes fraction was more abundant in the olive leaf EO, the most abundant compound was *neo-*intermedeol, an oxygenated sesquiterpene whose relative abundance accounted for up to 17.7%. 4-Terpineol (15.3%), an oxygenated monoterpene, and the apocarotenoid dihydrodehydro-β-ionone (3.6%) followed among the most represented compounds in this EO’s composition. The non-terpene derivatives, detected as the third chemical class in order of relative concentration (15.0%) in this sample, were mostly represented by non-terpene aldehydes: The most relevant presence was detected for (*E*,*E*)-2,4-decadienal (3.5%), nonanal (2.8%), and (*E*)-2-decenal (1.1%). The published olive leaf EO compositions evidence a significant variability due to both the analyzed varieties and the geographical area of origin [30,31,32,33]; moreover, the olive leaves of the present study are a blend of two varieties.

The lemon leaf EO was rich in oxygenated monoterpenes, which accounted for over 48%: the most abundant ones were neryl acetate (15.3%), nerol (8.0%), geranial (5.1%), and geranyl acetate (5.1%). The single most abundant compound in the present EO composition was β-caryophyllene (16.5%), representing over half of the sesquiterpene hydrocarbons fraction, which was the second most quantitatively important chemical class of compounds (27.9%). β-Bisabolene and bicyclogermacrene followed in the latter group of compounds, with relative abundances of 3.6 and 3.5%, respectively. Limonene (6.3%) and β-pinene (5.8%) were detected as the most abundant monoterpene hydrocarbons (14.7%) in this EO, whilst spathulenol (3.2%) and caryophyllene oxide (2.7%) were the main oxygenated sesquiterpenes (7.2%). Among the reported EO compositions extracted from lemon leaves, great variability is evident from the literature, both in qualitative and quantitative terms. Leaves of lemon specimens from Crete Island (Greece) were mainly rich in limonene, but also the aldehydes and acetic esters of both nerol and geraniol were detected in relevant percentages [34]. The most abundant compound in the composition of an Indian lemon leaf EO was *(Z)*-sabinene hydrate (17.4%), followed by geraniol (14.6%) and α-pinene (4.3%) [35]. The composition of a leaf EO extracted from an Iranian lemon specimen was, instead, dominated by linalool, which represented over 30%, followed by geraniol (15.5%), α-terpineol (14.5%), and linalyl acetate (13.5%) [36]. The geographical area of origin of the samples clearly plays a major role in this organ’s EO composition.

The orange leaf EO was dominated (over 80% of the total composition) by oxygenated monoterpenes: The most abundant compounds detected for this class were linalool (34.3%) and its acetic ester (21.3%), followed by α-terpineol (13.8%), geranyl (5.5%), and neryl (3.1%) acetates. The only other relevant chemical class of volatiles detected for this EO was the monoterpene hydrocarbons, which accounted for 18.5%: Myrcene (4.5%), (*E*)-β-ocimene (4.4%), and β-pinene (3.9%) were the most abundant. The high relative abundances of linalool, linalyl acetate, and α-terpineol are consistent with literature reports for orange leaf EO obtained from different accessions [37,38,39,40].

### 2.2. Chemical Characterization of the Leaf Olive Oils

The quality characterization of olive oils obtained by means of the addition of citrus and olive leaves to the olives during the oil extraction has been investigated, taking into account not only the simple chemical parameters, such as total acidity and peroxide value, but also the bioactive compounds (chlorophylls, carotenoids, tocopherols, total polyphenols), as well as the antioxidant activity.

#### 2.2.1. Quality Parameters

Quality parameters, such as free acidity and peroxide number, together with spectrophotometric indices, are all valuable olive oil freshness indices. As reported in Table 2, even though the milled olives showed a high ripening index (4 on a scale of 7), all the olive oil samples had free acidity, peroxide values, and spectrophotometric indices within the maximum limits established by EU Regulation [41] for extra-virgin olive oil (EVOO).

Free acidity covered a range from 0.65 (olive LOO) to 0.80 g oleic acid/kg oil (lemon LOO), while the peroxide value never outreached 12 meq O_2_/kg oil, representing a good level of olive oil freshness.

In particular, the oil fortified with olive leaf exhibited the lower value of peroxides (7.2 meq O_2_/kg oil, *p* < 0.05), which are reactive oxygen species (ROS), known for having a key role in cellular physiology as they can react with DNA, proteins, or lipids to alter their normal functions [42].

The low values of K_232_, K_270_, and ∆K also confirmed the good overall quality of control and leaf olive oils.

#### 2.2.2. Phenolic Content, Free-radical scavenging capacity, Total Carotenoid, and Total Chlorophylls

The established health benefits of olive oils, due to their biological activities, required their quali-quantitative characterization with regard to their bioactive compound content. The total phenol content of all the olive oils was determined by Folin–Ciocalteau assay. As evidenced in data reported in Table 3, orange and olive leaf olive oils, together with the control, have the highest total phenols content, followed by the lemon leaf olive oil.

The sensory properties of olive oil are largely affected by its phenolic composition because these compounds have been associated with the bitter and pungent sensory notes of oil [43], so the oil samples have also been chemically characterized in terms of the compounds responsible for oil bitterness, evaluated spectrophotometrically at 225 nm (Intensity of Bitterness). The oil obtained by adding orange leaf to olives exhibited the highest value of IB (2.11), followed by olive leaf olive oil (1.95), lemon leaf olive oil (1.64), and control olive oil (0.85).

The effect of antioxidants on 2,2′-azino-di-(3-ethylbenzthiazoline sulphonate) (ABTS) radical scavenging was thought to be due to their hydrogen-donating ability, so the scavenging effects of the four olive oil extracts on ABTS were examined as a function of the different fortifying agents (*Olea europeae*, *Citrus limon* or *C. aurantium* leaf). The Free-radical scavenging capacity of the oil extracts was assessed also by 2,2-Diphenyl-1-picrylhydrazyl (DPPH) assay, in order to obtain more accurate information on their actual antioxidant power. In particular, using both the methodologies, orange and olive leaf olive oils showed a radical-scavenging activity comparable to that of the control, while the lemon leaf olive oil exhibited the lowest value, as expected because of its phenolic content.

Olive oil color is directly connected to the chlorophyll and carotenoid contents, and it has been proposed as a characterizing factor and as a quality index related to the oil extraction method and to olive variety [44,45]. In the studied oils, only chlorophyll showed significant differences among samples. The oil extracted with the addition of olive leaves exhibited the highest value for total chlorophyll, closely followed by the control, while the chlorophyll content of the citrus leaf olive oils ranged from 6.63 (orange leaf olive oil) to 7.97 mg/kg (lemon leaf olive oil). The major increase is probably due to the increased extracted “chlorophyll *a*” from the leaves that naturally leads to the formation of derivate products, particularly “pheophytin *a*” [46]. These compounds, together with carotenoids (i.e., lutein and β-carotene), have been shown to attribute a more intense, greener pigmentation and improve nutritional attributes, but they also act as prooxidants in the light, so these olive oils should be absolutely protected from light exposure during storage [47].

#### 2.2.3. Phenolic Compounds

Virgin olive oils contain different classes of phenolic compounds: Phenolic alcohols, aldehydes and acids, but also secoiridoids, lignans, and flavones. The concentrations of phenolic compounds were then determined in leaf olive oil samples, in comparison with the control EVOO. Tyrosol, vanillin and oleuropein were the most abundant phenolic compounds detected in the olive oil samples (Table 4).

The addition of leaves to the olives during extraction determined a significant increase in the vanillin content (more than +20%), whereas the tyrosol content showed an opposite trend, especially when orange leaves were used (about –40%).

The amounts of phenolic acids in the oils under investigation are in accordance with those previously reported in the literature [48], where concentrations of phenolic acids were reported in the range of 0.01–1.70 mg/kg for EVOOs. According to Rahmanian and co-workers [49], *p*-Coumaric acid was the predominant acid in all the leaf olive oil samples, which also showed a high content of caffeic acid.

Oleuropein aglycone (3,4-DHPEA-EA) was found in all the samples, but the highest concentration was detected in the olive oil extracted in the presence of olive leaf (+50% in comparison with the control).

#### 2.2.4. Tocopherols

Tocopherols are significant nutritional constituents and, among them, α- and γ-tocopherol represent the most effective lipid-soluble antioxidants. For this reason, the analysis of tocopherol content in the four olive oils was performed (Table 5).

In all the samples, α-tocopherol was the most represented isomer, with values always higher than 100 mg/kg. In contrast, δ-tocopherol was the least represented isomer (Table 5). Following leaf addition, both α- and δ-tocopherol showed an increasing trend, particularly as regards orange and lemon LOOs. The levels of the γ-isomer remained instead unaffected by the leaf addition (Table 5).

### 2.3. Control’s and LOOs’ Headspaces

Overall, 30 compounds were identified among the headspaces of the analyzed extra-virgin olive oils (EVOOs): Their complete compositions are reported in Table 6.

The headspace sampling of the control obtained by a blend of ‘Leccino’ and ‘Moraiolo’ cultivars revealed only five compounds, out of which four are non-terpene derivatives. In this chemical class, (*E*)-2-hexenal and nonanal are the most abundant; the former represented over 80% of the total composition, followed by the latter, whose relative abundance accounted for 5.3%. Thus, the non-terpene fraction is dominated by aldehydes. The predominant presence of (*E*)-2-hexenal is reported as a desirable characteristic of high-quality EVOOs [50]. The C6 saturated and unsaturated aldehydes are synthesized through the lipoxygenase pathway, which uses the polyunsaturated fats as precursors; in particular, a reaction commonly reported for high-quality EVOOs involves the isomerization of *(Z)*-3-hexenal, derived from the enzymatic action of hydroperoxide lyases, to (*E*)-2-hexenal by means of an isomerase [50]. The (*E*)-2 aldehyde forms are, indeed, more stable to oxidation compared to their *(Z)*-3 counterparts [50]. The volatile organic compounds (VOCs) released in the EVOOs’ headspaces are responsible for the perceived aroma of these products and their preference among consumers. (*E*)-2-Hexenal is reported as a bitter and astringent flavor, with green and fruity aroma notes, perceived as quite sharp in the mouth [51]; its presence is reported as dominant in ‘Leccino’ monovarietal EVOOs [50,52]. Nonanal aroma notes are reported as fatty and soapy, with a citrus-like flavor [50,53]. In the framework of a European Union-funded project (FLAIR-CT0046) aimed at analyzing the composition of monovarietal EVOOS, both the astringent and fruity flavors, with quite a sharp mouth perception, were reported as favorable notes among consumers [54,55]. The only detected sesquiterpene hydrocarbon, (*E*,*E*)-α-farnesene, although accounting for only 0.5%, is reported for the ‘Leccino’ EVOO headspace [52].

In the headspaces of the LOOs, although non-terpene derivatives were detected as the most abundant chemical class of compounds (over 75%), monoterpene hydrocarbons showed a relevant relative abundance: Whilst undetected in the control sample, they accounted for up to 21.5% in the olive leaf LOO headspace, with (*E*)-2-hexenal representing over half (51.7%) of its composition. Moreover, all the other compounds detected in the control’s headspace were identified in the olive leaf LOO sample, as well: Whilst (*E*)-2-hexenal and nonanal decremented, (*E*)-4,8-dimethylnona-1,3,7-triene and (*E*)-2-dodecene showed an increase. The notable presence of limonene, which accounted for over 17% in this sample’s headspace, although being detected in a relative concentration as low as 0.4% in the olive LOO, can be explained by a matrix effect: The lipophilic nature of the olive oil extracted and concentrated the more lipophilic VOCs from the olive leaves. A published study reported an increase in *(E)*-2-hexenal content in olive LOO, as well as (*Z*)-3-hexenol, (*E*)-2-hexenol, and 1-hexanol [56]. Compared to these results, the headspace of the present study showed an opposite behavior in terms of the relative abundance of (*E*)-2-hexenal, whilst for the other listed compounds, they were not even detected in the control sample. This could be explained by the varietal and blend differences of the analyzed samples, even though Di Giovacchino et al. [56] explained this increment as a result of the crushing of the olive leaves, which releases the chloroplast content. Compared to the results reported by Malheiro et al. [57], decanal and β-caryophyllene did not increase with the addition of olive leaves; moreover, they were not present in the control sample to begin with.

The addition of citrus leaves noticeably modified the composition of the olive oils’ headspaces: Monoterpene hydrocarbons became the most abundant compounds, accounting for over 96% in the orange LOO. Limonene showed the highest relative abundance in the headspace of both the lemon and sour orange leaf EVOO samples. The lemon LOO sample, though, exhibited a composition richer in oxygenated monoterpenes compared to the orange LOO; nerol, neral and their related acetic esters were detected in noteworthy relative amounts in the lemon sample, whilst they were not evidenced at all (with the exception of geranial, which accounted for 0.1%) in the orange LOO.

Compositional differences were also evidenced among the monoterpene hydrocarbons: β-pinene was found with a 12.2% relative presence in the lemon sample, whilst in the sour orange it only accounted for 0.7%; moreover, γ-terpinene, (*E*)-β-ocimene, and α-pinene were all over 1% in the lemon sample, whilst they were lower in the sour orange one. These compositional changes showed an opposite trend compared to the essential oils; this might be due to a lower affinity of the EVOO lipophilic matrix towards the oxygenated monoterpenes detected in the orange leaf EO (linalool, α-terpineol, and linalyl acetate).

### 2.4. Sensory Quality

According to the International Olive Council standards for sensory evaluation, the merceological classification of olive oils is based on the evaluation of both ‘negative’ and ‘positive’ attributes [58]. Among the positive sensory attributes, fruity notes should be identified in the EVOO, whereas negative ones (sensory defects) cannot be present [14].

No sensory defects were detected in any of the analyzed oil samples. Moreover, the analysis of the overall descriptors showed that all the samples had a positive evaluation for whole pleasantness and fruity character, confirming the good overall quality of these leaf olive oils. According to European legislation [41], these olive oils could be considered extra-virgin olive oils (EVOO), due to their lack of defects and fruitiness value and due to their chemical features (see Table 3). This indicates that the addition of citrus or olive leaves during olive extraction did not cause the loss of the positive sensory attributes characteristic of EVOO, regardless of the botanic origin of the leaves.

The lemon LOO showed the highest intensity of odor among all the samples, with high fruity notes, while the olive LOO had the lowest intensity of odor, which was characterized by a high vegetal scent.

**Figure 1:**
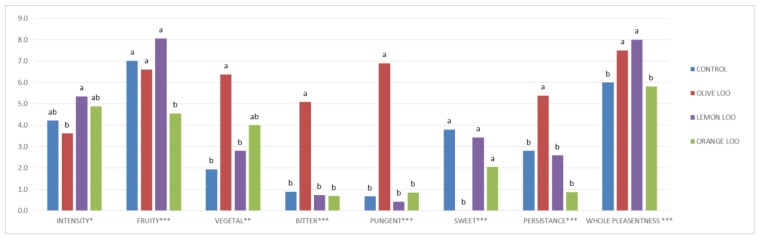
Main sensorial descriptors of control, olive leaf olive oil (olive LOO), orange leaf olive oil (orange LOO), and lemon leaf olive oil (lemon LOO) ranked by panel components. For the same descriptor, significant differences (at *p* ≤ 0.05) are indicated by different letters.

As expected considering the phenolic content of the samples, while the addition of olive leaves determined a significant increase in bitter and pungent notes perceived by the panelists in comparison with the control oil, the presence of citrus leaves during extraction did not lead to the same results (see Figure 1).

**Figure 2:**
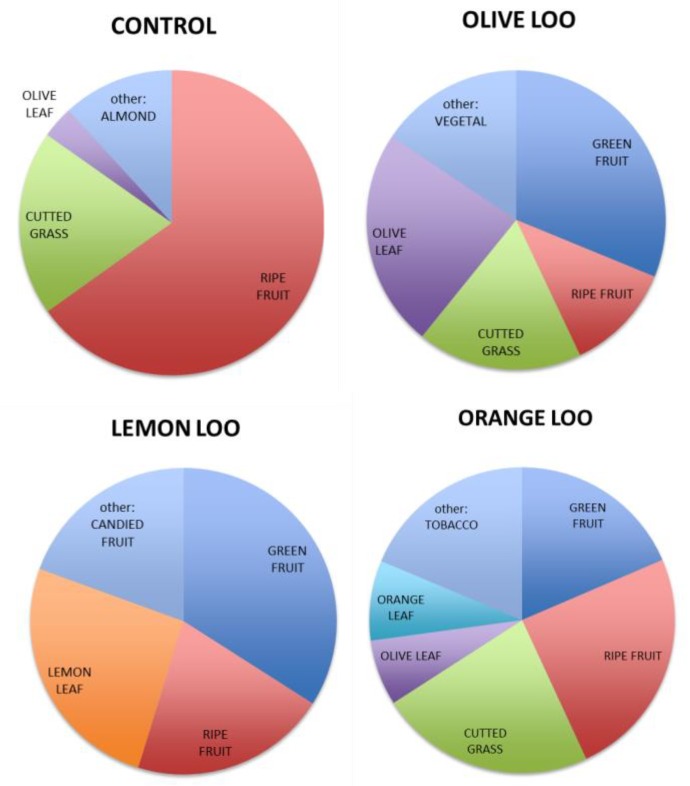
Qualitative smell profiles obtained by sensory analysis of control, olive leaf olive oil (olive LOO), lemon leaf olive oil (lemon LOO), and orange leaf olive oil (orange LOO).

Indeed, the notes linked to the phenolic content of the oils obtained using citrus leaves were not different from the control. From a qualitative point of view, the specific smell profiles of the four olive oils appeared very different to each other (see Figure 2).

In the presence of leaves, the scent of ripe fruit, generally associated with an overripe raw material or quite an old olive oil, was interestingly mitigated in all the LOOs. Furthermore, the addition of leaves during olive oil extraction seemed to increase the smell complexity, thus also improving the recognizability of the oils obtained. Thereafter, the lemon and olive LOOs also showed the highest whole pleasantness (Figure 1), while the highest smell complexity was associated to the orange LOO.

## 3. Discussion

Due to the strong health evidence related to some of the olive’s phenolic compounds, EU legislation [59] has enabled the use of health claims on extra-virgin olive oil labels, “*Olive oil polyphenols contribute to the protection of blood lipids from oxidative stress*.” This health claim is allowed only for olive oil containing at least 0.25 mg/g of hydroxytyrosol and its derivatives (e.g., oleuropein complex and tyrosol) [60]. Among the antioxidant compounds generally associated with olive oil, oleuropein has several pharmacological properties, including antioxidant, anti-inflammatory, antiatherogenic, anticancer, antimicrobial, antiviral, and cardioprotective [61], and the present results confirm that olive leaf addition during oil extraction may represent a valid approach to increase the oleuropein concentration (Table 4). This enrichment was due to the presence in large amounts of oleuropein in olive leaves [18], which did not occur in citrus leaves, thus explaining the lack of increment in oleuropein amounts in orange and lemon LOOs (Table 4).

As widely reported in literature [47,49], olive leaves show high-added value, being an excellent source of compounds with biologic properties, particularly phenolic compounds, exhibiting a strong protective effect against oil oxidation [18].

In this context, according to Malheiro et al. [47], since the inclusion of several antioxidant compounds extracted from the leaves in olive oil lead to a considerable increase in the oxidative stability and nutritional quality, this technology could be profitably applied as a way to improve olive oil quality, also when ripe olives are used. Indeed, the chemical composition of olives changes with ripening, in a way that is cultivar-dependent. In particular, a decline in phenolic compounds and green pigmentation occurs with ripening, resulting in a decrease in olive oil stability [62].

In contrast, vitamin E is always present in the leaf, independently of the species, explaining the general increase in vitamin E found in the fortified oils (Table 5). The main isomer represented is α-tocopherol, which is well-known for its ability to act as a hydroperoxyl radical scavenger, therefore it plays an important role in protecting the organism against oxidative damage [63]. Even though, for many years, α-tocopherol has been considered as the most active form of vitamin E present in humans [64] preventing the detrimental effects of free radicals as agents of plasma lipid peroxidation, γ-tocopherol has recently been acknowledged as the “other” vitamin E important for human health, capable of trapping peroxynitrites [65]. The maintenance of γ-tocopherol quantities in oils after leaf addition (Table 5) is in agreement with what was previously found for two Tunisian varieties [18]. As reported in the literature [19,21,66,67,68], cryomaceration of vegetal byproducts by means of solid carbon dioxide can be profitably applied to improve the green extraction of bioactive compounds, favoring the next mass transfer processes in solid–liquid extraction. The method is based on the evidence that the volume occupied by the same amount of water in the solid state is greater than that in the liquid phase, thus the addition of a cryogen to vegetal matrices induces intracellular water freezing, and the consequent laceration of cellular membranes (cellular break) induces the immediate diffusion in the liquid phase of many cellular compounds [67].

In this context, on the basis of the obtained results, this study proposes an efficient and innovative green procedure, using solid carbon dioxide as cryogen, to improve the nutraceutical and sensory qualities of olive oils extracted from overripe raw material. By means of the addition of citrus or olive cryomacerated leaves milled together with olives during oil extraction, it was possible to obtain LOOs characterized by a higher content of antioxidants compared to the control sample, and their specific composition varied as a function of the botanic origin of the leaves. In the adopted experimental conditions, the highest oleuropein concentration was detected in the olive oil extracted in the presence of olive leaf (+50% in comparison with the control).

Moreover, a daily consumption of about 15 mL (one spoonful) of citrus LOO, which showed the highest α-tocopherol concentration, could be sufficient to fulfill the dietary recommendation for vitamin E [69].

The organoleptic profiles of the LOOs were also profitably improved in terms of whole pleasantness as well as smell complexity, when compared with the control.

## 4. Materials and Methods

### 4.1. Plant Materials

The *Olea europaea* leaves belonged to Moraiolo and Leccino cultivars and came from a private company, while the *Citrus* leaves were collected at the Monumental Charterhouse of Pisa (Calci, Italy).

The olive oils enriched by the addition of *Citrus* and *Olea* leaves, as well as the control (not flavored), were produced from Moraiolo and Leccino olive varieties provided by a private company located in Tuscany (San Miniato, Pisa-Italy) during the 2018/2019 crop season (Table 7).

### 4.2. Essential Oil Extractions

All the extractions were performed on roughly cut plant material (a blend of dried leaves for *Olea europaea* L. of ‘Leccino’ and ‘Moraiolo’ varieties, fresh leaves for *Citrus × aurantium* L. and *Citrus limon* (L.) Osbeck). A standard Clevenger-type apparatus was used for all the EOs; the extraction time was 2 h for all the samples. The obtained EOs were dehydrated over anhydrous magnesium sulfate, then diluted to 0.5% in HPLC grade *n*-hexane prior to GC–MS injection. The injections were performed immediately after each extraction.

### 4.3. Headspace Solid Phase Microextractions (HS-SPME)

The headspaces of the extra-virgin olive oil (EVOO) samples (control and with the addition of leaves) were sampled by solid phase microextraction. The adsorption of the volatile analytes was performed with a Supelco polydimethylsiloxane fiber assembly (100 μm coating thickness, St. Louis, MO, USA) preconditioned according to the manufacturer instructions. After the equilibration time, the septum of each vial is perforated by the holder (syringe), then the fiber is exposed to the headspace of the sample for 30 min at room temperature. Once the sampling is complete, the fiber is retracted into the holder and directly injected in the GC–MS apparatus for separation and analysis. All the SPME sampling and desorption conditions were identical for all the EVOOs. Furthermore, blanks were performed before each first SPME extraction and randomly repeated during each series. Quantitative comparisons of relative peak areas were performed between the same chemicals in the different samples.

### 4.4. Gas Chromatography–Mass Spectrometry Analyses and Peak Identification

Gas chromatography–electron impact mass spectrometry (GC–EIMS) analyses were performed with an Agilent 7890B gas chromatograph (Agilent Technologies Inc., Santa Clara, CA, USA) equipped with an Agilent HP-5MS (Agilent Technologies Inc., Santa Clara, CA, USA) capillary column (30 m × 0.25 mm; coating thickness 0.25 μm) and an Agilent 5977B single quadrupole mass detector (Agilent Technologies Inc., Santa Clara, CA, USA). The oven temperature program was set to rise from 60 °C to 240 °C at 3 °C/min. The set temperatures were as follows: Injector temperature, 220 °C; transfer-line temperature, 240 °C. The carrier gas was He, at 1 mL/min flow. For the essential oils, the injection volume was set at 1 μL. The acquisition was performed with the following parameters: Full scan, with a scan range of 35–300 *m/z*; scan time: 1.0 s; threshold: 1 count. The identification of the constituents was based on the comparison of their retention times (t_R_) with those of pure reference samples and their linear retention indices (LRIs), which were determined relatively to the t_R_ of a series of *n*-alkanes. The detected mass spectra were compared with those listed in the commercial libraries NIST 14 and ADAMS, as well as in a homemade mass-spectral library, built up from pure substances and components of known oils and in MS literature data [19 and literature therein].

### 4.5. Leaf Olive Oil Extraction

Leaves were cryomacerated with solid carbon dioxide (1:1 in weight) overnight and then directly added (3% in weight) to olives before milling, as previously described [18,19]. The extraction runs were carried out using a micro oil mill equipped with a two-phase decanter (Spremioliva C30, produced by “Toscana Enologica Mori”, Tavernelle Val Di Pesa, Florence, Italy) able to mill 25–35 kg of olives per hour, following the protocol developed by [66].

### 4.6. Sample Collection

For each thesis, 10 kg of leaves as well as 300 kg of olives (ratio Moraiolo:Leccino = 1:1) were collected the day before the beginning of the experimental runs and immediately stored at 5 °C in a fruit cold storage room until the extraction process. An aliquot of fruits and leaves was immediately processed in order to proceed with the chemical-physical characterization.

For each oil extraction experimental run, an aliquot of oil was immediately stored in dark glass bottles at 12 °C in an inert atmosphere until the sensory and chemical analyses. Each analysis was performed one day before the extraction in three replicates. In Table 8, the main samples collected during the experimental research are reported, together with their codes.

### 4.7. Leaf Olive Oil Chemical Analyses

#### 4.7.1. Quality Parameters

Free fatty acids (FFA), peroxide value (PV), and spectrophotometric indices (K_232_, K_270_ and ∆K) were determined according to the Official EU analytical methods described in the Regulation EEC/2568/91 and later modifications [41].

#### 4.7.2. Analysis of Phenolic Content

Phenolic compounds were extracted from the oil sample as previously described [70] using methanol/water (70:30, *v/v*) in the extraction phase; then, the extracts were stored under inert atmosphere at −20 °C until use. The determination of the total phenols was performed according to the Folin–Ciocalteau colorimetric method, using gallic acid as standard [71].

#### 4.7.3. HPLC Analysis of Phenolic Compounds

Qualitative and quantitative analyses were performed by reverse-phase HPLC (RP-HPLC) [72]. As reported by Tarchoune et al. [18], twenty microliters of extract were injected into a Waters model 515 HPLC system fitted with a 3.9 mm × 150 mm Nova-Pak C18 column (Waters, Milford, MA, USA). Detection was conducted at 280 nm using a Waters 2487 dual UV–visible detector. Mobile phase A contained 98% water and 2% acetic acid, and mobile phase B contained 68% water, 30% acetonitrile and 2% acetic acid. A linear gradient of 10 to 95% mobile phase B was run for 90 min at 1 mL/min. The identity of the phenolic acids was confirmed by chromatography on HPLC with authentic standards (Sigma Chemical Co., St. Louis, MO, USA), and quantification was performed using a standard curve in the range of 20 to 200 ng of standard mixtures containing gallic, protocatechuic, *p*-hydroxybenzoic, chlorogenic, vanillic, caffeic, syringic, *p*-coumaric, ferulic, tyrosol, hydroxytyrosol, vanillin, and oleuropein. Chromatogram analysis was performed by the software Millennium 32 (Waters).

#### 4.7.4. Free-radical scavenging capacity (FRSC)

The free-radical scavenging activity of oil samples was measured following the DPPH assay (FRSC_DPPH_) and the ABTS assay (FRSC_ABTS_). The DPPH assay is based on the abilities of the antioxidants present in the extracts to scavenge the free radical 2,2-Diphenyl-1-picrylhydrazyl in comparison with that of Trolox; it was performed following the method reported by Brand-Williams et al. [73]. The ABTS assay was performed following the method reported by Sgherri et al. [74], using the radical cation ABTS. The radical solution was prepared as previously described [75], and a Trolox dose–response curve in the 0.2–1.5 mM range was used. The antioxidant activity was expressed as Trolox equivalent Free-radical scavenging capacity (TEAC) per mL of extract.

#### 4.7.5. Intensity of Bitterness (IB) Determination

The IB was determined following the method previously described by Gutiérrez Rosales et al. [76]: Bitter components were extracted from 1.00 ± 0.01 g of oil samples, by means of octadecyl (C18) disposable extraction columns (6 mL) (J.T. Baker Chemical Company, Phillipsburg, NJ, USA); then, absorbance was recorded at 225 nm.

#### 4.7.6. Pigment Determination

Total carotenoids and chlorophylls were colorimetrically determined, at 470 and 670 nm respectively, according to the method previously described by Mínguez-Mosquera et al. [77].

#### 4.7.7. Extraction and Detection of Tocopherols (Vitamin E)

Tocopherol extractions were performed in the dark as previously reported by Sgherri et al. [65] and according to the method of Gimeno et al. [78].

Tocopherol isoforms (α-, β-, γ-, and δ-) were determined by isocratic RP-HPLC using a Shimadzu apparatus (model LC-20AD) with an electrochemical detector (Metrohm model 791, Varese, Italy) equipped with a glassy carbon electrode and LC Solution software (Shimadzu) for the integration of peaks, as reported by Tarchoune et al. [18]. Detection was performed according to Galatro et al. [79] at +0.6 V and at 25 °C, with a Nova Pak C-18 4 μm column (3.9 × 150 mm), as reported by Tarchoune et al. [18]. The extracts were eluted with 95% methanol containing 20 mM LiClO_4_ at a flow rate of 1 mL/min. For the identification and quantification of peaks, a calibration curve was prepared using standard mixtures of α-, β-, γ-, and δ- tocopherol provided by Sigma (Milan, Italy) in the range of 25 to 75 ng, as reported by Tarchoune et al. [18].

### 4.8. Leaf Olive Oil Sensory Analysis

The quantitative descriptive analysis of the oil samples was performed by a panel of 10 trained assessors included in the “expert panel” of the Department of Agriculture, Food and Environment (DAFE) of the University of Pisa, according to the internal procedure for assessor selection and training [80]. The sensory characterization followed the methods described by [41,58]. To better describe the organoleptic profile of the oil samples, the panel was provided with a technical evaluation sheet, reported in a previous paper [19].

The sensory profiles were defined on the basis of, (i) the first-order descriptors of color, flavoring, and taste and (ii) the hedonic parameter related to the evolutionary state and whole pleasantness [19]. The panelists ranked the flavored oil samples on a scale from 0 (no perception, minimum) to 9 (maximum) to evaluate the intensity of each parameter. The tasting was carried out under the previously described conditions [80,81].

Furthermore, panelists were asked to indicate some specific olfactory descriptors with the aim of better characterizing the quality and complexity of the smell profiles of the different oils.

### 4.9. Statistical Analysis

The results are the means ± SD of three independent experiments. The significance of differences among means was determined by one-way ANOVA (CoStat, Version 6.451, CoHort Software, Pacific Grove, CA, USA). Comparisons among means were performed by the Bartlett’s X2 corrected test (*p* < 0.05).

## Figures and Tables

**Table 1:** Complete compositions of essential oils extracted from leaves of *Olea europaea* L., *Citrus × aurantium* L. and *Citrus limon* (L.) Osbeck.

Compounds	Chemical Class ^a^	L.R.I. ^b^	Relative Abundance (%)		
			Olive Leaf Essential Oil (EO)	Lemon Leaf EO	Orange Leaf EO
furfural	NT	839	0.3	- ^c^	-
(*E*)-3-hexen-1-ol	NT	853	1.4	-	-
1-hexanol	NT	871	0.3	-	-
heptanal	NT	901	0.1	-	-
α-pinene	MH	941	-	0.2	0.3
benzaldehyde	NT	963	0.6	-	-
(*Z*)-2-heptenal	NT	963	0.1	-	-
3-ethenyl pyridine	N-NT	975	1.8	-	-
sabinene	MH	976	-	0.7	0.6
1-octen-3-ol	NT	980	0.1	-	-
β-pinene	MH	982	-	5.8	3.9
6-methyl-5-hepten-2-one	NT	985	0.2	0.2	-
2-pentyl furan	NT	993	0.5	-	-
myrcene	MH	993	-	0.2	4.5
octanal	NT	1001	0.2	-	-
(*E*,*E*)-2,4-heptadienal	NT	1012	1.0	-	-
α-terpinene	MH	1018	1.1	-	-
*p*-cymene	MH	1027	0.2	-	-
limonene	MH	1032	0.4	6.3	1.4
1,8-cineole	OM	1034	-	1.2	-
(*Z*)-β-ocimene	MH	1042	-	0.2	2.1
phenyl acetaldehyde	NT	1045	0.2	-	-
(*E*)-β-ocimene	MH	1052	-	1.1	4.4
γ-terpinene	MH	1062	0.3	0.2	-
(*E*)-2-octenal	NT	1064	0.2	-	-
*cis*-sabinene hydrate	OM	1070	0.9	0.2	-
*n*-octanol	NT	1071	0.9	-	-
benzyl formate	NT	1080	0.2	-	-
terpinolene	MH	1088	0.7	-	1.3
rosefuran	OM	1093	-	0.2	-
*trans*-sabinene hydrate	OM	1095	0.8	-	-
linalool	OM	1101	0.7	4.0	34.3
nonanal	NT	1102	2.8	-	-
1,3,8-*p*-menthatriene	MH	1113	0.3	-	-
*trans*-*p*-mentha-2,8-dien-1-ol	OM	1121	1.5	-	-
*trans*-limonene oxide	OM	1141	0.7	-	-
*trans*-*p*-menth-2-en-1-ol	OM	1143	0.5	-	-
citronellal	OM	1155	-	1.5	-
(*E*,*Z*)-2,6-nonadienal	NT	1156	-	-	0.1
*iso*neral	OM	1171	-	0.3	-
4-terpineol	OM	1178	15.3	0.2	0.2
*p*-cymen-8-ol	OM	1183	0.8	-	-
*iso*geranial	OM	1184	-	0.3	-
α-terpineol	OM	1189	1.1	1.2	13.8
methyl salicylate	NT	1192	1.4	-	-
*cis*-piperitol	OM	1195	1.0	-	-
*trans*-piperitol	OM	1207	0.4	-	-
nerol	OM	1230	0.6	8.0	2.6
neral	OM	1240	-	4.2	-
geraniol	OM	1257	0.6	1.7	-
linalyl acetate	OM	1259	-	-	21.3
(*E*)-2-decenal	NT	1260	1.1	-	-
geranial	OM	1271	-	5.1	-
limonen-10-ol	OM	1290	0.3	-	-
thymol	OM	1292	0.5	-	-
dihydroedulan	NT	1293	0.7	-	-
theaspirane I	AC	1298	2.7	-	-
(*E*,*E*)-2,4-decadienal	NT	1316	3.5	-	-
citronellyl acetate	OM	1350	-	0.4	-
α-terpinyl acetate	OM	1352	-	-	0.2
eugenol	PP	1358	2.1	-	-
neryl acetate	OM	1366	-	15.3	3.1
(*E*)-β-damascenone	AC	1384	2.9	-	-
geranyl acetate	OM	1385	-	5.1	5.5
γ-dihydroionone	AC	1396	0.8	-	-
β-longipinene	SH	1398	1.9	-	-
β-caryophyllene	SH	1420	0.4	16.5	0.3
dihydrodehydro-β-ionone	AC	1424	3.6	-	-
*trans*-α-bergamotene	SH	1438	-	2.1	-
(*E*)-*iso*eugenol	PP	1448	1.7	-	-
(*E*)-geranyl acetone	AC	1455	0.6	-	-
α-humulene	SH	1456	-	1.6	-
(*E*)-β-ionone	AC	1490	1.6	-	-
bicyclogermacrene	SH	1495	-	3.5	-
(*Z*)-α-bisabolene	SH	1504	-	0.3	-
β-bisabolene	SH	1509	-	3.6	-
δ-cadinene	SH	1524	-	0.3	-
*cis*-sesquisabinene hydrate	OS	1545	1.4	-	-
germacrene d-4-ol	OS	1575	-	0.7	-
spathulenol	OS	1576	-	3.2	-
caryophyllene oxide	OS	1581	-	2.7	-
α-cedrene epoxide	OS	1585	1.4	-	-
1,10-*di*-*epi*-cubenol	OS	1614	0.8	-	-
humulane-1,6-dien-3-ol	OS	1619	1.6	-	-
α-cadinol	OS	1654	-	0.4	-
*neo*intermedeol	OS	1660	17.7	-	-
*epi*-α-bisabolol	OS	1686	-	0.2	-
drimenol	OS	1755	1.0	-	-
kaurene	DH	2043	1.1	-	-
Monoterpene hydrocarbons			2.8	14.6	18.6
Oxygenated monoterpenes			25.7	48.9	81.0
Sesquiterpene hydrocarbons			2.4	27.9	0.3
Oxygenated sesquiterpenes			23.8	7.3	-
Diterpene hydrocarbons			1.1	-	-
Apocarotenoids			12.9	-	-
Nitrogen compounds			1.8	-	-
Phenylpropanoids			3.9	-	-
Other non-terpene derivatives			15.0	0.2	0.1
Total identified (%):			89.3	98.8	100.0

^a^ Chemical classes of compounds: Monoterpene hydrocarbons (MH), oxygenated monoterpenes (OM), sesquiterpene hydrocarbons (SH), oxygenated sesquiterpenes (OS), diterpenes hydrocarbons (DH), apocarotenoids (AC), nitrogen compounds (N-NT), phenylpropanoids (PP), non-terpene derivatives (NT); ^b^ Linear retention indices on a HP-5MS column; ^c^ Not detected.

**Table 2:** Chemical characterization of the control, orange leaf olive oil (orange LOO), lemon leaf olive oil (lemon LOO), olive leaf olive oil (olive LOO) and legal limits for extra-virgin olive oil (EVOO) [41]. Each value represents mean ± standard deviation (*n* = 3).

	Reference EVOO	Control	Olive Leaf Olive Oil (LOO)	Lemon LOO	Orange LOO
Free Acidity (g oleic acid/kg oil)	≤ 0.80	0.74 ^b^ ± 0.01	0.65 ^c^ ± 0.01	0.80 ^a^ ± 0.01	0.66 ^c^ ± 0.01
Peroxide Value (meq O_2_/kg oil)	≤ 20.00	8.90 ^a^ ± 0.17	7.20 ^b^ ± 0.01	9.00 ^a^ ± 0.01	9.00 ^a^ ± 0.01
K_232_	≤ 2.50	1.98 ^a^ ± 0.01	1.92 ^ab^ ± 0.01	1.86 ^b^ ± 0.01	1.92 ^ab^ ± 0.01
K_270_	≤ 0.22	0.14 ± 0.01	0.10 ± 0.01	0.12 ± 0.01	0.15 ± 0.01
ΔK	≤ 0.10	0.00	0.00	0.00	0.00

Within each row, significant differences (at *p* ≤ 0.05) are indicated by different letters.

**Table 3:** Total phenolic content, intensity of bitterness, Free-radical scavenging capacity, total carotenoid and total chlorophylls of the control, orange leaf olive oil (Orange LOO), lemon leaf olive oil (Lemon LOO), and olive leaf olive oil (Olive LOO). Each value represents mean ± standard deviation (*n* = 3).

	Control	Olive LOO	Lemon LOO	Orange LOO
Total phenols Content (TPC) (ppm gallic acid)	144 ^a^ ± 4	150 ^a^ ± 1	115 ^b^ ± 3	142 ^a^ ± 1
Intensity of Bitterness (IB)	0.85 ^d^ ± 0.01	1.95 ^b^ ± 0.02	1.64 ^c^ ± 0.01	2.11 ^a^ ± 0.01
Free-radical scavenging capacity (FRSC_ABTS_) (μmol TEAC/mL)	0.40 ^a^ ± 0.01	0.36 ^a^ ± 0.01	0.20 ^b^ ± 0.01	0.38 ^a^ ± 0.01
Free-radical scavenging capacity (FRSC_DPPH_) (μmol TEAC/mL)	0.34 ^a^ ± 0.02	0.29 ^a^ ± 0.01	0.15 ^b^ ± 0.02	0.28 ^a^ ± 0.02
Total carotenoid (TC) (mg/kg of lutein)	4.62 ± 0.01	4.58 ± 0.02	4.20 ± 0.01	4.20 ± 0.01
Total chlorophylls (TCH) (mg/kg of pheophytin)	9.93 ^b^ ± 0.01	10.04 ^a^ ± 0.01	7.97 ^c^ ± 0.02	6.63 ^d^ ± 0.01

Within each row, significant differences (at *p* ≤ 0.05) are indicated by different letters.

**Table 4:** Composition of phenolic alcohols, aldehyde and acids (mg/kg oil) in control, olive leaf olive oil (olive LOO), lemon leaf olive oil (lemon LOO), and orange leaf olive oil (orange LOO). Each value represents mean ± standard deviation (*n* = 3).

	Control	Olive LOO	Lemon LOO	Orange LOO
**Phenolic Alcohols**				
Hydroxytyrosol	0.057 ± 0.002	0.062 ± 0.020	0.063 ± 0.008	0.081 ± 0.010
Tyrosol	2.730 ^a^ ± 0.136	2.553 ^ab^ ± 0.203	2.157 ^b^ ± 0.135	1.574 ^c^ ± 0.081
**Phenolic Aldehydes**				
Vanillin	0.293 ^b^ ± 0.011	0.354 ^a^ ± 0.026	0.345 ^a^ ± 0.027	0.373 ^a^ ± 0.038
**Phenolic Acids**				
Vanillic acid	0.142 ^a^ ± 0.009	0.124 ^ab^ ± 0.008	0.088 ^c^ ± 0.006	0.105 ^bc^ ± 0.002
Caffeic acid	0.008 ^b^ ± 0.001	0.012 ^a^ ± 0.002	0.012 ^a^ ± 0.001	0.013 ^a^ ± 0.001
Syringic acid	0.102 ^a^ ± 0.012	0.028 ^b^ ± 0.007	0.017 ^b^ ± 0.001	0.007 ^b^ ± 0.002
*p*-Coumaric acid	0.001 ^b^ ± 0.000	0.141 ^a^ ± 0.008	0.154 ^a^ ± 0.014	0.173 ^a^ ± 0.015
Ferulic acid	0.026 ± 0.003	0.028 ± 0.005	0.026 ± 0.003	0.034 ± 0.002
**Secoiridoids**				
Oleuropein aglycone	17.021 ^b^ ± 0.534	26.577 ^a^ ± 1.701	12.957 ^c^ ± 0.600	16.028 ^b^ ± 0.345

Within each row, significant differences (at *p* ≤ 0.05) are indicated by different letters.

**Table 5:** Composition of vitamin E, expressed as δ-, γ-, and α-tocopherol (mg/kg oil) in control, olive leaf olive oil (olive LOO), lemon leaf olive oil (lemon LOO), and orange leaf olive oil (orange LOO). Each value represents mean ± standard deviation (*n* = 3).

Vitamin E	Control	Olive LOO	Lemon LOO	Orange LOO
δ-tocopherol	0.53 ^b^ ± 0.02	0.48 ^b^ ± 0.19	0.74 ^a^ ± 0.29	1.10 ^a^ ± 0.29
γ-tocopherol	3.38 ± 0.51	2.93 ± 0.40	3.37 ± 0.04	3.60 ± 0.15
α-tocopherol	117.09 ^c^ ± 3.33	122.37 ^bc^ ± 2.33	137.87 ^a^ ± 4.22	132.24 ^ab^ ± 4.58

Within each row, significant differences (at *p* ≤ 0.05) are indicated by different letters.

**Table 6:** Complete compositions of headspaces (HS) of control, olive leaf olive oil (Olive LOO), lemon leaf olive oil (Lemon LOO), and orange leaf olive oil (Orange LOO).

Compounds	Chemical Class ^a^	L.R.I. ^b^	Relative Abundance (%)			
			**Control**	**HS Olive LOO**	**HS Lemon LOO**	**HS Orange LOO**
(*E*)-2-hexenal	NT	856	80.8	51.7	- ^c^	-
α-pinene	MH	941	-	-	1.1	0.8
β-pinene	MH	982	-	-	12.2	0.7
myrcene	MH	993	-	-	-	2.1
limonene	MH	1032	-	17.8	41.5	91.9
(*Z*)-β-ocimene	MH	1042	-	3.7	-	-
(*E*)-β-ocimene	MH	1052	-	-	1.5	0.7
γ-terpinene	MH	1062	-	-	1.6	0.1
*n*-octanol	NT	1071	-	-	-	0.1
terpinolene	MH	1088	-	-	0.3	0.1
linalool	OM	1101	-	-	0.9	1.0
nonanal	NT	1102	5.3	2.5	0.6	-
(*E*)-4,8-dimethylnona-1,3,7-triene	NT	1116	3.9	12.4	-	-
citronellal	OM	1155	-	-	0.7	-
*iso-*geranial	OM	1184	-	-	0.1	-
α-terpineol	OM	1189	-	-	0.3	0.1
decanal	NT	1204	-	-	0.1	0.1
(*E*)-2-dodecene	NT	1205	9.5	11.4	-	-
octyl acetate	NT	1214	-	-	-	0.1
nerol	OM	1230	-	-	5.7	-
neral	OM	1240	-	-	12.3	-
geraniol	OM	1257	-	-	2.2	-
linalyl acetate	OM	1259	-	-	-	1.6
geranial	OM	1271	-	-	15.0	0.1
eugenol	PP	1358	-	0.2	-	-
neryl acetate	OM	1366	-	-	1.7	0.1
geranyl acetate	OM	1385	-	-	0.4	0.2
β-caryophyllene	SH	1420	-	-	1.1	0.1
*trans*-α-bergamotene	SH	1438	-	-	0.1	-
(*E*,*E*)-α-farnesene	SH	1507	0.5	0.4	0.4	-
Monoterpene hydrocarbons			-	21.5	58.2	96.4
Oxygenated monoterpenes			-	-	39.3	3.1
Sesquiterpene hydrocarbons			0.5	0.4	1.6	0.1
Phenylpropanoids			-	0.2	-	-
Other non-terpene derivatives			99.5	78.0	0.7	0.3
Total identified (%)			100.0	100.0	99.8	99.9

^a^ Chemical classes of compounds: Monoterpene hydrocarbons (MH), oxygenated monoterpenes (OM), sesquiterpene hydrocarbons (SH), phenylpropanoids (PP), non-terpene derivatives (NT); ^b^ Linear retention indices on a HP-5MS column; ^c^ Not detected.

**Table 7:** Olive fruit characterization.

Parameter	Olive Fruit Characterization
Ripeness Index (0:7)	4.0 ± 0.2
Average Weight (g)	1.50 ± 0.01
Average Volume (cm^3^)	1.4 ± 0.1
Water Content (%)	48.43 ± 0.02
Oil Content (% dry matter)	19.70 ± 0.04

**Table 8:** Sample codes used during the experimental research.

Sample Code	Description
Olive leaf EO	Essential oil extracted from *Olea europaea* L. leaves
Lemon leaf EO	Essential oil extracted from *Citrus limon* L. leaf
Orange leaf EO	Essential oil extracted from *Citrus × aurantium* L. leaves
Control	Extra-virgin olive oil
Olive LOO	Oil obtained by adding *Olea europaea* leaves to olive during olive oil extraction
Lemon LOO	Oil obtained by adding *Citrus limon* L. leaves to olive during olive oil extraction
Orange LOO	Oil obtained by adding *Citrus × aurantium* L. leaves to olive during olive oil extraction
